# Athlete Mental Health and Wellbeing During the Transition into Elite Sport: Strategies to Prepare the System

**DOI:** 10.1186/s40798-024-00690-z

**Published:** 2024-03-09

**Authors:** Vita Pilkington, Simon Rice, Lisa Olive, Courtney Walton, Rosemary Purcell

**Affiliations:** 1 Centre for Youth Mental Health, The University of Melbourne, Melbourne, Australia; 2 Elite Sport and Mental Health, Orygen, 35 Poplar Road, Melbourne, Australia; 3 School of Psychology, Deakin University, Geelong, VIC Australia; 4 IMPACT Institute for Mental and Physical Health and Clinical Translation, Deakin University, Geelong, Australia; 5 School of Psychological Sciences, The University of Melbourne, Melbourne, Australia

## Abstract

The transition into elite-level sport can expose young athletes to risk factors for mental ill-health, including increased performance expectations, stressors associated with becoming increasingly public figures, and changes in lifestyle demands, such as diet, training loads and sleep. Successful integration into elite-level sport requires athletes to quickly adapt to these newfound challenges and the norms and culture of the new sport setting, while developing relationships with teammates, coaches, and support staff. Despite these demands, the mental health experiences of athletes transitioning into elite-level sport have been largely neglected in sport psychology literature. This is reflected in the dearth of programs for supporting mental health during this career phase, particularly relative to retirement transition programs. In this article, we offer a preliminary framework for supporting athletes’ mental health during the transition into elite-level sport. This framework is based on holistic, developmental, and ecological perspectives. Our framework outlines a range of recommendations for promoting mental health and preventing mental ill-health, including individual-level, relational, sport-level, and sociocultural-level strategies. Key recommendations include preparing athletes for the challenges they are likely to face throughout their athletic careers, highlighting athletes’ competence earlier in their careers, developing supportive relationships in the sport setting, and fostering psychologically safe sporting cultures. Supporting mental health from earlier in the athletic career is likely to promote athletes’ overall wellbeing, support enjoyment and retention in sport, and encourage help-seeking.

## Key Points

- The mental health of athletes transitioning to retirement has gained considerable attention, but less attention has been granted to athletes’ mental health during the transition *into* elite-level sport.
- The transition into elite sport involves exposure to a range of risk factors for mental ill-health and requires athletes to adjust rapidly for successful integration into the new sport setting.
- This framework includes recommendations for supporting athletes’ mental health and wellbeing during the transition into elite-level sport.

## Introduction

Elite athletes—or athletes who undertake specialised training and compete at national or international levels [1, 2]—experience a range of sport-related transitions over the course of their athletic careers [3]. Sport-related transitions experienced by elite athletes include sport specialisation, progression to higher levels of performance, selection and deselection, pre- and post-major competition, injury and recovery, relocation, and retirement [4, 5]. While transitions to retirement have been considered from a mental health perspective, little attention has been paid to supporting mental health during the transition *into* elite sport settings.

Transitioning out of sport due to planned or unplanned/involuntary retirement (often due to serious/chronic injury or performance decline) is associated with adjustment difficulties, loss of identity and mental ill-health [6, 7]. The latter includes elevated rates of depression, suicidality, anxiety, low self-esteem, and substance use [6, 8–12]. Given the importance of athlete adjustment when transitioning out of elite sport, sporting organisations are increasingly investing in programs to support athletes during this career phase, recognising that this transition is a *process* rather than a single-occasion activity [7, 13]. Such programs are critical given the comparatively young age at which most elite athletes ‘retire’ relative to their community counterparts, often necessitating career and identity ‘reinvention’.

The lack of focus applied to athletes’ transitions or onboarding *into* elite sport settings is surprising, given that this is a phase associated with a range of potentially destabilising factors. Here we advocate for a framework to better support and protect athlete mental health during the transition into elite sport environments. Based on principles of early intervention, we argue for an explicit focus on supporting elite athlete mental health via:

1. Equipping athletes with adequate knowledge about elite sport and challenges associated with transitioning into these settings;
2. Equipping athletes with mental health literacy and self-management skills to promote early recognition of, and response to, possible adjustment difficulties or mental ill-health associated with the transition; and
3. Equipping sporting organisations (including coaches and team managers) with strategies for promoting healthy athlete development, identity formation, and mental health from the earliest point of their professional elite careers.

## Demands Associated with the Transition into Elite-Level Sport

Athlete outcomes following career transitions are impacted by the type of transition (i.e., reasons underlying the transition and its level of predictability), the way the transition is appraised (i.e., favourably vs. unfavourably), internal characteristics (e.g., resilience, optimism), available resources (e.g., social support) and the coping strategies implemented when responding to the transition [14–16]. Transitions that are unpredictable and/or unaligned with an athlete’s goals can precipitate mental ill-health, particularly in the absence of sufficiently effective coping strategies and resources [8, 17, 18].

Transitioning to higher levels of competition likely aligns with the goals of most athletes. Such progression indicates increasing athletic proficiency, often providing access to benefits, such as greater financial and public recognition. Nonetheless, transitioning into elite competition also presents complex demands that can contribute to mental ill-health [19]. Such demands include the rapid introduction to more regimented structures (e.g., changes to diet, sleep, training load) and to established teams, including coaching staff, high performance support staff and teammates. These changes occur alongside exposures to known risk factors for mental ill-health, such as relocation, extended periods of travel, reduced connection to social networks, and increased performance pressure [5, 19–24]. Athletes may also experience stressors related to greater selection competition, risk of de-selection, greater living and financial independence, and newfound demands related to becoming an increasingly public figure, including public scrutiny, social media abuse, pressure to be a role model, and sponsorship requirements [20, 25, 26]. While navigating these changes, athletes in the onboarding phase are *also* tasked with learning about—and quickly adapting to—the systems, processes, norms, and culture within the new sporting environment.

Former athletes reflecting on their career progression have reported both positive and negative impacts of transitioning into higher levels of competition [23]. Positive impacts included feeling more competent in their athletic abilities and greater commitment to their sport as their careers advanced. However, they also described intensive training loads, insufficient time with loved ones and challenges in the sporting context (e.g., conflict with coaches, frustration associated with lacking autonomy about selection and organisational decision-making).

Increased performance expectations are significant sources of stress for many athletes transitioning to higher levels of competition [19, 21, 22, 24, 27, 28]. Elevated performance expectations can be internal, imposed by athletes themselves, or external, imposed by coaches, teammates and/or caregivers [27–29]. Higher expectations can result in perceived inadequacy, including normative comparison with athlete peers and fear of not meeting others’ expectations, in addition to self-doubt and increased pre-competition performance anxiety [21, 22, 29]. Relatedly, maladaptive sporting role perfectionism may be worsened by needing to ‘earn’ playing time at the elite level through consistently strong performance [22]. This pressure can be compounded by sponsorship and financial pressures in elite-level sport [19].

Due to feeling underprepared for the transition in terms of higher performance demands and changes associated with the new environments, athletes entering higher levels of competition describe feelings of uncertainty and lacking control [21]. Some describe that unfamiliarity with the team, club and organisational culture feed into poor clarity regarding behavioural expectations [27]. Relational issues within the sport setting may be experienced during this phase, with some athletes reporting conflict with coaches and difficulty coping with negative performance feedback [22, 24, 27, 30].

As a consequence of dedicating more time and commitment towards their sporting roles, athletes’ life balance in other domains (e.g., social and academic/vocational pursuits outside sport) can be compromised during this transition (e.g., [28]), which risks athlete identity foreclosure, or the commitment to the athletic role and identity at the expense of exploring other aspects of the identity [31, 32]. While a strong athletic identity can protect against burnout and increase enjoyment in—and commitment to—the sporting role [33, 34], athlete identity foreclosure is a risk factor for mental ill-health, particularly among injured, retiring or recently retired athletes [35–37].

Finally, the age at which most athletes transition into elite-level sport overlaps significantly with the peak age of onset for mental ill-health [38, 39]. Throughout this paper, ‘elite youth athletes’ generally includes those aged 12–17 years, as recently recommended by Walton and colleagues [40]. However, age of entry into elite sport (including both professional sporting codes and Olympic level sports) can differ significantly according to sport type, and in some circumstances, athletes transitioning into elite sport may comprise those aged under 12 years or 18 years or older [41]. We argue that athletes’ vulnerability to mental ill-health due to their age and developmental stage, coupled with the demands of the transition into elite sport, warrants a focus on how best this transition can be supported.

The changes and stressors experienced during the transition into elite sport can lead to adjustment difficulties, mental ill-health, loss of enjoyment in sport, emotional and physical exhaustion, overtraining, injury, and burnout [22, 30, 42]. Despite this, barriers to help-seeking—including mental health stigma, lack of awareness about support resources, and concerns about the consequences of seeking help (e.g., de-selection)—may be particularly salient to athletes entering into elite sporting environments [43, 44].

Despite the varied challenges associated with the transition from pre-elite to elite sport and associated risk of experiencing mental ill-health, current emphasis in sport psychology literature is biased towards consideration of mental health *during* the athletic career and *after* the athletic career, rather than the entry into elite settings.

## The Sport Setting as a Key Context for Athletes’ Development

Given the time, commitment and dedication required for an athlete to reach and maintain elite status, the sport setting is a space for not only athlete *talent* development, but also for the development of the person as a whole, including their social relationships, character, sense of self and worldview [42, 45]. Elite youth athletes typically experience sport-related transitions in parallel with key developmental tasks, such as identity exploration, autonomy development, and developing future life goals [42, 46, 47]. Athletes transitioning into elite sport often recognise that their career progression comes at the expense of other areas of life and strengthens their commitment to the athletic role and identity [19]. While athlete retirement transition programs recommend preventing athletic identity foreclosure throughout the athletic career [4, 48], little attention to preventing this risk has occurred during the transition *into* elite sport.

## Existing Programs for Supporting Athletes Transitioning into Elite Sport

We are aware of only three programs for supporting athletes’ transitions into elite sport. Larsen and Alferman [49] developed educational workshops highlighting challenges soccer players may experience when preparing to move to the professional level, assisting athletes to develop strategies for preparing for the transition (e.g., coping strategies, goal setting, and psychological skill development). A program evaluation indicated that players reported several benefits, including access to transparent information about transition-related challenges and developing relationships within the club before the transition (via incorporating role models such as senior players and coaching staff into the program). Coaches also reported benefits related to individualised goal setting activities, which helped players maintain motivation towards pursuing their career goals [49].

Another promising program, designed by Pummell and colleagues, was implemented and evaluated at an international high-performance tennis centre in the United Kingdom [50]. Elite junior-level tennis players participated in 10 workshops, which involved discussion about the upcoming transition into senior-level elite tennis. This included equipping players with realistic expectations about the transition (e.g., higher performance expectations and requirement for greater responsibility, independence, and discipline), and videos of senior elite players reflecting on their transition experiences and helpful coping strategies. An evaluation indicated increased knowledge among players about transition demands and readiness to cope with the transition [50].

Cupples and colleagues [51] developed a program for youth Rugby League players transitioning into high-performance environments. The program aimed to support effective transitions via upskilling coping strategies, such as problem-solving, support seeking, and breathing techniques. An evaluation found increased task-based coping (e.g., problem solving, seeking coach feedback on selection and training errors) and decreased use of avoidance coping, but found no changes in wellbeing or support-based coping. To our knowledge, no programs specifically designed to target the promotion of, and support for, mental health during the entry into elite sport exist, and there are no frameworks to inform the development of such programs.

## A Framework for Supporting Athlete Mental Health During the Transition into Elite Sport

**Fig. 1:**
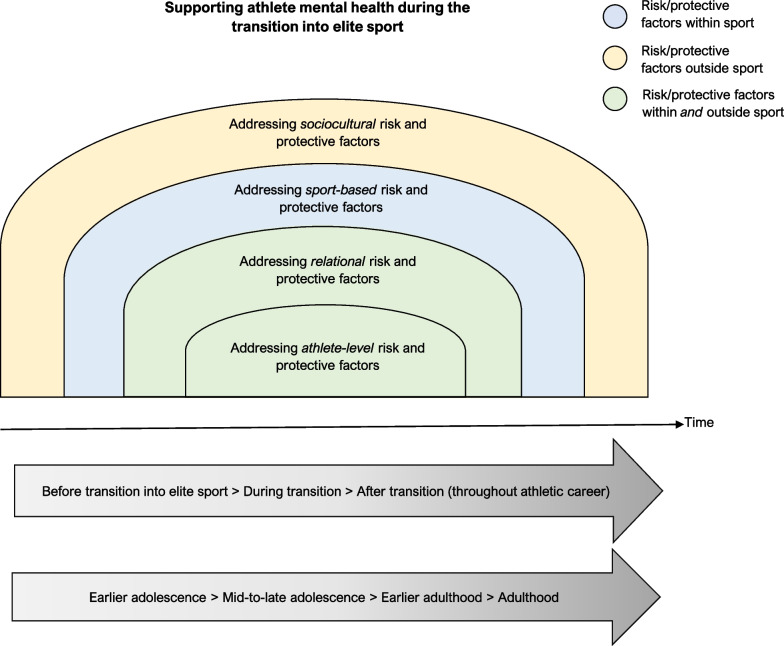
Framework for supporting athlete mental health during the transition into elite sport (and beyond)

In the general population, favourable outcomes associated with prevention and early intervention for mental ill-health are well-established [52, 53]. We propose that onboarding programs that provide athletes with the knowledge and skills to navigate the transition into elite sport will lead to more favourable mental health, wellbeing, performance, and retention outcomes in the elite sport system [47, 48]. Athletes will be better placed to respond to both sport-related and non-sport related stressors and transitions if they are equipped with effective coping strategies and skills to build resilience, *and* if the sporting environment they are entering recognises these challenges and provides the necessary resources to support this transition [4, 10]. Figure 1 presents an overarching framework for supporting mental health and wellbeing in athletes transitioning into elite sport settings, which is accompanied by recommendations throughout Sects. “Strategies for Addressing Individual-Level Mental Health Risk and Protective Factors”–“Strategies for Addressing Sociocultural Mental Health Risk and Protective Factors”.

Our framework is predicated on the duty of care that elite sporting organisations have in developing and maintaining safe and supportive athlete environments. The framework is informed by holistic and developmental [45, 54] and ecological perspectives [48, 55], most notably, works from Wylleman and Stambulova [42, 45, 54, 56]. The framework is also informed by a recent narrative review by Walton and colleagues [40], and Sabato and colleagues’ recommendations for supporting elite youth athlete physical and emotional health [2]. The holistic and developmental perspective—largely informed by Wylleman and Stambulova’s works—conceptualizes the athlete as a *whole person*, rather than only a sportsperson [15], recognising that healthy development includes vocational/academic, psychosocial, and financial domains, and that changes in each of these occur over the course of athletes’ lives and careers [42, 45, 54]. Within an ecological perspective, a range of risk and protective factors may influence athlete mental health at the individual, social, sporting, and cultural/societal levels [48]. This approach helps to build accountability for protecting mental health *throughout* the relevant sport ecology, rather than placing the onus on an individual athlete.

This framework extends previously proposed ecological models for supporting athlete mental health (e.g., [48, 57]) by acknowledging that risk and protective factors for mental health overlap with major life transitions, including developmental changes and athletic career progression. Taken together, these approaches inform a set of practical recommendations for supporting athlete mental health during the transition into elite sport. These recommendations focus on enhancing protective factors for mental health at the individual athlete level, as well as the relational, sporting/organisational, and sociocultural levels. These strategies can target factors both *within* the sport system (e.g., athlete-coach relationships, individual-level coping strategies for sport-related stressors) and *outside* the sport system (e.g., athlete relationships with family and friends, maintaining non-athletic identities).

### Strategies for Addressing Individual-Level Mental Health Risk and Protective Factors

Strategies to strengthen athletes’ resilience to the stressors associated with the entry into elite-level sport and to promote positive mental health outcomes include providing opportunities for autonomy, highlighting competence, preventing identity foreclosure, and preparing athletes for possible sport-related stressors [4, 10, 48]. Many of these strategies are included as key components of retirement transition programs. We argue that these protective factors for mental health can be implemented in earlier phases of athlete careers as foundational components for mental health.

Developmental considerations are necessary here given the young age at which many athletes transition into elite sport (often early-to-mid adolescence, if not younger). While providing athletes with full autonomy is not always feasible, possible strategies include asking athletes for feedback about key organisational decisions or outcomes, co-developing personalised training plans based on athletes’ self-rated strengths, weaknesses, and career goals, and adjusting training schedules to accommodate educational, vocational, or familial commitments.

Assisting coaching staff to better recognise and emphasise athletes’ competence during the transition into elite sport is also recommended. Highlighting competence and providing opportunities for autonomous decision-making from early in the elite sporting career can promote positive sport-related outcomes (e.g., enjoyment in the sporting role, motivation to engage in sport) and wellbeing outcomes (e.g., self-esteem, decision-making skills) [58–60].

Preventing athletic identity foreclosure early in an athlete’s career is also critical [4, 21, 23, 28, 31], since this is a known risk factor for mental ill-health [35–37]. This can be facilitated in onboarding programs via structured and individualised career guidance that encourages athletes to consider future career goals outside their athletic roles, as well as informal strategies, such as coaches and other key staff taking an interest in athletes’ educational/vocational pursuits and life interests outside of sport.

Given the number of changes associated with the transition into elite sport, it is recommended that onboarding programs provide athletes with transparent information about the challenges they are likely to experience during the transition (and potentially throughout their careers) and how these can be mitigated. This should ideally involve the voices of current and/or former athletes who have experienced and managed these challenges (recognising that many challenges, such as performance pressure, negative feedback from coaches or public scrutiny are largely unavoidable). Equipping athletes with adaptive coping skills and self-management strategies to build resilience should be a critical component of these programs. Evidence-based strategies in this regard include healthy self-talk (with an emphasis on self-compassion and recognising athletes’ intrinsic value as people), use of problem-solving techniques, and grounding or mindfulness techniques [48, 61, 62].

Despite best prevention efforts, some athletes will nonetheless experience adjustment difficulties and mental ill-health over the course of their careers [39, 62]. Provision of mental health literacy programs for athletes transitioning into elite sport is warranted. These should focus on (1) promoting early recognition of mental ill-health, burnout, and adjustment difficulties, (2) encouraging help-seeking, and (3) providing practical advice regarding where and how to access support. As recommended by Gorczynski and colleagues [63], these mental health literacy programs should be tailored to the athletes’ stage of development and should be informed, where possible, by the organisation’s contextual factors (e.g., available resources, existing support pathways, identified needs among individuals within the organisation). Further, the ongoing evaluation of mental health literacy programs is essential to ensure these programs are deemed acceptable, appropriate, and are meeting their primary aims [63].

### Strategies for Addressing Relational Mental Health Risk and Protective Factors

Meaningful and well-functioning relationships, both inside and outside sport, are another key protective factor for mental health [64, 65]. Sporting organisations should provide opportunities for onboarding athletes to develop supportive relationships with teammates, coaching staff, high performance staff and others in the environment, including via mentoring programs or similar. From the onboarding stage onwards, coaching and other high performance staff should engage in genuine conversations with athletes that promote connection (e.g., asking about their lives and interests outside sport) to ensure that athletes feel intrinsically valued as *people*, rather than only valued for their skills and performance [48, 66]. This can assist with building psychological safety (see Sect. “Strategies for Addressing Sociocultural Mental Health Risk and Protective Factors”) in the sports environment, enabling athletes to establish a sense of belonging, while feeling safe to ask questions [67, 68]. It is also recommended that sporting organisations facilitate the development of social networks and opportunities for communication between coaching staff and athletes’ caregivers (or other key supports), as both coaches and caregivers can be well-placed to recognise changes in an athlete’s mood or behaviour that reflect early indications of mental ill-health (e.g., social withdrawal, negative comments about weight or shape, loss of enjoyment in sport) [69]. Accordingly, the delivery of mental health literacy programs to caregivers prior to the athletes’ transition into the new sport setting is recommended to support the recognition of transition difficulties and/or mental health symptoms and to upskill caregivers in having effective conversations about these experiences [40].

Induction programs can also provide coaching and support staff with optimal strategies for communicating with newly entering athletes. Here there is opportunity to highlight the benefits of *mastery-oriented* coaching styles, where there is a focus on personal improvement and displays of effort and persistence in the face of setbacks. This can be seen as distinct to *ego-oriented* coaching styles, focusing on social comparison with other athletes [70].

### Strategies for Addressing Sport-Level Mental Health Risk and Protective Factors

At the sport (or organisational) level, it is recommended that sporting organisations are responsive to their safeguarding responsibilities, offer encouragement for athlete help-seeking, and routinely monitor athlete mental health. Further, clearly identifying *who* in the organisation is responsible for organising and implementing specific mental health strategies is advisable to ensure these activities are prioritised on an ongoing basis. This may be facilitated by allocating mental health ‘champions’ in the organisation who are well-informed about the organisation’s mental health strategy, can serve as points-of-contact for mental health-related questions or concerns, and hold key responsibilities in ensuring any mental health activities/programs are delivered as planned [69]. Crucially, these ‘champions’ should be clearly identifiable among athletes and staff.

In addition to ensuring appropriate safeguarding policies and procedures *exist,* information about reporting processes and pathways (in response to bullying, harassment, discrimination, or abuse) should be provided to athletes entering any elite sport system. Onboarding athletes will be less knowledgeable about the systems, processes and procedures for reporting adverse events, and likely less confident reporting and seeking support, relative to more experienced athletes [71]. Onboarding programs should provide mandatory safeguarding information to ensure that athletes understand *how* they can report concerns [72, 73]. Safeguarding information should also be disseminated to athletes’ caregivers and across the organisation to coaching, high performance, executive/leadership, and administration staff.

Similarly, sporting organisations should ensure that athletes transitioning into the new sport setting—in addition to staff within the organisation—receive adequate opportunities for seeking support for mental ill-health, and that information about pathways for help-seeking is readily available from early in the athletic career. Given athletes transitioning into these environments may be hesitant to disclose mental health problems due to confidentiality concerns and possible ramifications of seeking support (e.g., loss of selection, being viewed as weak) [43], information concerning confidentiality should be highlighted in communications about opportunities for support.

It is also recommended that athletes transitioning into elite sport are screened for symptoms of mental ill-health before, during, and after the transition to facilitate early identification and intervention. Where possible, sport-sensitised tools for detecting mental ill-health, psychological distress, and perceived psychological safety should be used (e.g., [74–76]). Screening practices should be accompanied by clear processes for responding to elevated scores, offering pathways for discussion and support where needed [77].

### Strategies for Addressing Sociocultural Mental Health Risk and Protective Factors

Addressing sociocultural risk and protective factors for mental health is another essential component of a sporting organisation’s overall mental health strategy that should be prioritised from the onboarding process onwards. Here we highlight the importance of developing cultures that are ‘psychologically safe’ [78], reducing mental health stigma, and recognising and embracing diversity within the organisation.

Sport settings are often characterised as valuing toughness and stoicism. While these values can be helpful on-field, when applied rigidly to all situations, they can risk contributing to mental health stigma and barriers to help-seeking (e.g., concerns about being viewed as weak or others responding negatively to mental ill-health disclosures) [43, 48]. Sport teams, clubs, and organisations are responsible for developing cultures that are psychologically safe [68]. Psychologically safe environments are characterised by interpersonal trust, mutual respect, and acceptance of mistakes and differences among individuals within the environment [78, 79]. Improving psychological safety has been shown to enhance team cohesion, learning, innovation and performance outcomes in a range of high-performance organisational contexts [80]. There is increasing recognition that psychological safety can also support mentally healthy sport settings [67, 68, 74].

Sporting teams, clubs, and organisations are encouraged to *normalise* mental ill-health among their members, as this can promote psychological safety and facilitate help-seeking [43, 81]. Normalising mental ill-health can be facilitated by providing opportunities for open discussions about mental health, encouraging leadership to share mental health challenges they have experienced, encouraging individuals in the sport setting to respond to disclosures in supportive and non-judgmental ways, and framing mental health help-seeking as an important strategy for maintaining one’s *overall* health and wellbeing. Tackling mental health stigma and promoting openness about mental ill-health—including how to initiate conversations and have safe conversations with others—can be facilitated by mental health literacy programs that offer training in how to communicate about mental ill-health (e.g., Ahead of the Game) [82].

Given that individuals from diverse backgrounds (e.g., according to race/ethnicity, sexuality, gender expression) are more likely to experience discrimination and harassment, including in sporting contexts [83], sporting organisations should ensure that the diversity of staff and athletes is recognised and valued [69]. This may be facilitated by promoting meaningful conversations about diverse identities to better understand these experiences, seeking to reflect diversity across staff, providing opportunities for athletes and staff to engage in culturally meaningful practices and celebrations, and directly engaging with those from diverse backgrounds about their preferences regarding how their identities are discussed and shared among others within the organisation.

On a broader scale, sports may also be able to impact the broader public on issues surrounding mental health stigma and embracing diversity. For example, reducing mental health stigma via campaigns involving prominent sportspeople talking about their personal experiences of mental ill-health can be effective, particularly since elite athletes are commonly viewed by the public (and youth athletes) as strong, resilient, successful role-models [84]. Sporting organisations can also publicly address unacceptable public behaviour directed towards athletes that may contribute to mental ill-health, such as harassment, racism, or discrimination.

## Conclusion

The transition into elite sport is associated with a range of stressors. If left unchecked or unaddressed, these may contribute to psychological distress or mental ill-health, which in turn may impact an athlete’s performance and ability to achieve their career goals. The youth athlete career phase is a particularly vulnerable period for mental ill-health, given the upheaval in many athletes’ physical environments, social relationships, training and lifestyle demands, and performance expectations [19, 22]. This paper seeks to address the balance in attention to transitions within elite sport, shining a light on the entry into elite sport settings, and the opportunities available via structured induction/onboarding programs. We argue that this career phase represents a key *opportunity* to provide athletes with the foundational components for supporting their mental health and building resilience to manage the demands and rigors of elite sport. We note that given the nascency of literature in this field, this framework will warrant revision when increased evidence is available about athletes’ mental health needs during the transition into elite sport, as well as evidence-based strategies for supporting mental health during this career transition.

## Data Availability

Data sharing is not applicable to this article, as no datasets were generated or analysed in the preparation of this article.
